# Neuromuscular function and fatigue resistance of the plantar flexors following short-term cycling endurance training

**DOI:** 10.3389/fphys.2015.00145

**Published:** 2015-05-13

**Authors:** Martin Behrens, Matthias Weippert, Franziska Wassermann, Rainer Bader, Sven Bruhn, Anett Mau-Moeller

**Affiliations:** ^1^Institute of Sport Science, University of RostockRostock, Germany; ^2^Institute of Exercise Physiology and Public HealthRostock, Germany; ^3^Department of Orthopaedics, University Medicine RostockRostock, Germany

**Keywords:** explosive voluntary strength, maximal voluntary strength, H-reflex, V-wave, M-wave

## Abstract

Previously published studies on the effect of short-term endurance training on neuromuscular function of the plantar flexors have shown that the H-reflex elicited at rest and during weak voluntary contractions was increased following the training regime. However, these studies did not test H-reflex modulation during isometric maximum voluntary contraction (iMVC) and did not incorporate a control group in their study design to compare the results of the endurance training group to individuals without the endurance training stimulus. Therefore, this randomized controlled study was directed to investigate the neuromuscular function of the plantar flexors at rest and during iMVC before and after 8 weeks of cycling endurance training. Twenty-two young adults were randomly assigned to an intervention group and a control group. During neuromuscular testing, rate of torque development, isometric maximum voluntary torque and muscle activation were measured. Triceps surae muscle activation and tibialis anterior muscle co-activation were assessed by normalized root mean square of the EMG signal during the initial phase of contraction (0–100, 100–200 ms) and iMVC of the plantar flexors. Furthermore, evoked spinal reflex responses of the soleus muscle (H-reflex evoked at rest and during iMVC, V-wave), peak twitch torques induced by electrical stimulation of the posterior tibial nerve at rest and fatigue resistance were evaluated. The results indicate that cycling endurance training did not lead to a significant change in any variable of interest. Data of the present study conflict with the outcome of previously published studies that have found an increase in H-reflex excitability after endurance training. However, these studies had not included a control group in their study design as was the case here. It is concluded that short-term cycling endurance training does not necessarily enhance H-reflex responses and fatigue resistance.

## Introduction

Strength and endurance training are commonly used in physical training and rehabilitation programs. While a lot of studies have investigated and described neural and muscular adaptations to strength training (Aagaard et al., 2002; Del Balso and Cafarelli, 2007; Andersen and Aagaard, 2010; Ekblom, 2010), there are only a few studies available that have analyzed the effect of short-term (several weeks) endurance training on neural plasticity at the spinal and supraspinal level at rest and during isometric maximum voluntary contraction (iMVC) (Vila-Cha et al., 2012b; Zghal et al., 2014).

The electrically evoked Hoffmann reflex (H-reflex) and the volitional wave (V-wave) can be used to analyze intervention-induced modulations at the spinal level (Aagaard et al., 2002; Zehr, 2002). These evoked potentials are elicited by electrical stimulation of the posterior tibial nerve in the popliteal fossa and their amplitudes can be recorded in the soleus muscle (SOL). The H-reflex assesses the excitability of α-motoneurons via the Ia afferent pathway (Schieppati, 1987) whereas the V-wave is a measure of the descending neural drive from the α-motoneurons to the muscle (Aagaard et al., 2002).

Cross-sectional studies have revealed that stretch reflexes and H-reflexes elicited at rest are higher in athletes engaged in long-term (several years) endurance training than in athletes engaged in long-term power training (Kyrolainen and Komi, 1994a,b; Maffiuletti et al., 2001). Furthermore, longitudinal studies indicate that even short-term endurance training is able to modulate stretch reflex and H-reflex excitability of SOL (Perot et al., 1991; Vila-Cha et al., 2012b). It has been argued that the increased H-reflex after endurance training is the result of an altered responsiveness of the α-motoneuron pool to the Ia afferent volley and/or changes in presynaptic inhibition of Ia afferents (Vila-Cha et al., 2012b).

However, Perot et al. (1991) have not included a control group in their study design in order to compare the results of the endurance training group to individuals without the training stimulus. The authors reported that 75% of the investigated subjects showed an increased stretch reflex and H-reflex excitability after endurance training while in the remaining subjects no change or even a decrease in these parameters was evident. The study performed by Vila-Cha et al. (2012b) compared the effect of two different training regimes, i.e., 3 weeks of either endurance training on a cycle ergometer or strength training, on neuromuscular function of the plantar flexors. Thus, this study was lacking a control group without any systematic training stimulus as well. Furthermore, although data of the cited studies indicate that the H-reflex at rest and during weak voluntary contractions is increased in response to short-term endurance training (Perot et al., 1991; Vila-Cha et al., 2012b), nothing is known about H-reflex modulation during iMVC. Even though it has been shown that V-wave responses of SOL and cortical voluntary activation of the knee extensors were unchanged following short-term endurance training (Vila-Cha et al., 2012b; Zghal et al., 2014), H-reflex modulation during iMVC is of particular interest because it has been revealed that endurance athletes possess a greater iMVC strength compared to sedentary subjects (Lattier et al., 2003). Therefore, it is not unlikely that endurance training alters the contribution of the Ia afferent pathway to the muscle activity during iMVC.

In addition, fatigue resistance following a period of endurance training was previously tested by sustained isometric contractions using a defined percentage of iMVC strength until task failure. With this approach it has been shown that time to task failure increases following endurance training (Vila-Cha et al., 2012a,b). However, the time to task failure does not provide information regarding neural and muscular contributions to improved resistance to fatigue. Therefore, we applied the same dynamic fatigue protocol, i.e., with the same load, repetitions and range of motion, before and after the training period to assess the changes in neuromuscular function of the plantar flexors. With this approach we wanted to find out if endurance training is able to reduce the performance decrements associated with fatigue.

Accordingly, we analyzed the effect of an 8-week cycling endurance training period on neuromuscular function of the plantar flexors in this randomized controlled study. In particular, isometric explosive and maximum voluntary strength of the plantar flexors, normalized muscle activity of the plantar flexors and tibialis anterior muscle (TA) as well as electrically evoked spinal reflex responses (H-reflex at rest and during iMVC; V-wave) of SOL and contractile properties of the plantar flexors were analyzed. In addition selected variables, representing neural and muscular function of the plantar flexors, were again tested after a defined fatigue protocol with the same load, repetitions and range of motion before and after the training period.

We hypothesized that cycling endurance training would increase the normalized H-reflex at rest and during iMVC. Furthermore, we expected a reduction of the fatigue-induced performance decrements with regard to neural and muscular factors in the training group.

## Materials and methods

### Subjects

Twenty-two recreationally active subjects (moderate exercise <3 times per week, respectively, activities included swimming, strength training of the upper extremities and different sport games) with no history of neurological disorders or injuries volunteered for this study. The participants were randomly assigned to an intervention group and a control group using randomization by a computer-generated table of random numbers. The intervention group consisted of 11 subjects (6 females, 5 males, age: 24 ± 2 years, height: 172 ± 6 cm, body mass: 71 ± 8 kg), while 11 subjects were assigned to the control group (6 males, 5 females, age: 23 ± 2 years, height: 174 ± 6 cm, body mass: 70 ± 5 kg). Participants were not engaged in a systematic endurance training program in the 8 weeks prior to the study. The subjects were asked to avoid caffeine and alcohol consumption in the 24 h prior to the measurements. In addition, study participants were asked not to perform any strenuous exercise in the 48 h before the measurements. The study was conducted according to the declaration of Helsinki and was approved by the university's ethics committee.

### Endurance training

The subjects of the intervention group trained on a cycle ergometer (SP-SRP-3000, SportPlus, Hamburg, Germany) twice a week for 8 weeks with a total of 16 training sessions (at least 1 day rest between the training sessions). Each training session lasted approximately 60 min and was supervised by experienced instructors. The endurance training was performed in accordance with a previously published study on neuromuscular function of the plantar flexors following endurance training (Vila-Cha et al., 2012b). Exercise intensity was regulated according to the approach used by Dimeo et al. (1997) (with maximal heart rate estimated by 220 minus age in years). Individual exercise intensity during the training sessions was permanently monitored by heart rate monitors (Polar S810i, Kempele, Finland). The prescribed exercise intensities during the training are displayed in Table 1. The control group was asked to maintain their individual level of physical activities.

**Table 1:** **Exercise intensity as a percentage of estimated maximal heart rate (HR_max_) during the 8 weeks of endurance training**.

**Weeks**	**First training session**		**Second training session**	
	**Intensity**	**Duration (min)**	**Intensity**	**Duration (min)**
1	80% HR_max_	40	80% HR_max_	40
2	80% HR_max_	45	80% HR_max_	45
3	80% HR_max_	50	80% HR_max_	50
4	70% HR_max_	15	80% HR_max_	55
	90% HR_max_	10		
	70–80% HR_max_	20		
5	70% HR_max_	15	70% HR_max_	15
	90% HR_max_	10	90% HR_max_	10
	70–80% HR_max_	20	70–80% HR_max_	25
6	70% HR_max_	15	70% HR_max_	15
	90% HR_max_	15	90% HR_max_	15
	70–80% HR_max_	20	70–80% HR_max_	25
7	70% HR_max_	10	70% HR_max_	10
	90% HR_max_	10	90% HR_max_	15
	70–80% HR_max_	10	70–80% HR_max_	10
	90% HR_max_	10	90% HR_max_	10
	70–80% HR_max_	10	70–80% HR_max_	10
8	70% HR_max_	10	80% HR_max_	55
	90% HR_max_	15		
	70–80% HR_max_	10		
	90% HR_max_	10		
	70–80% HR_max_	10		

### Experimental procedure and fatigue protocol

**Figure 1:**
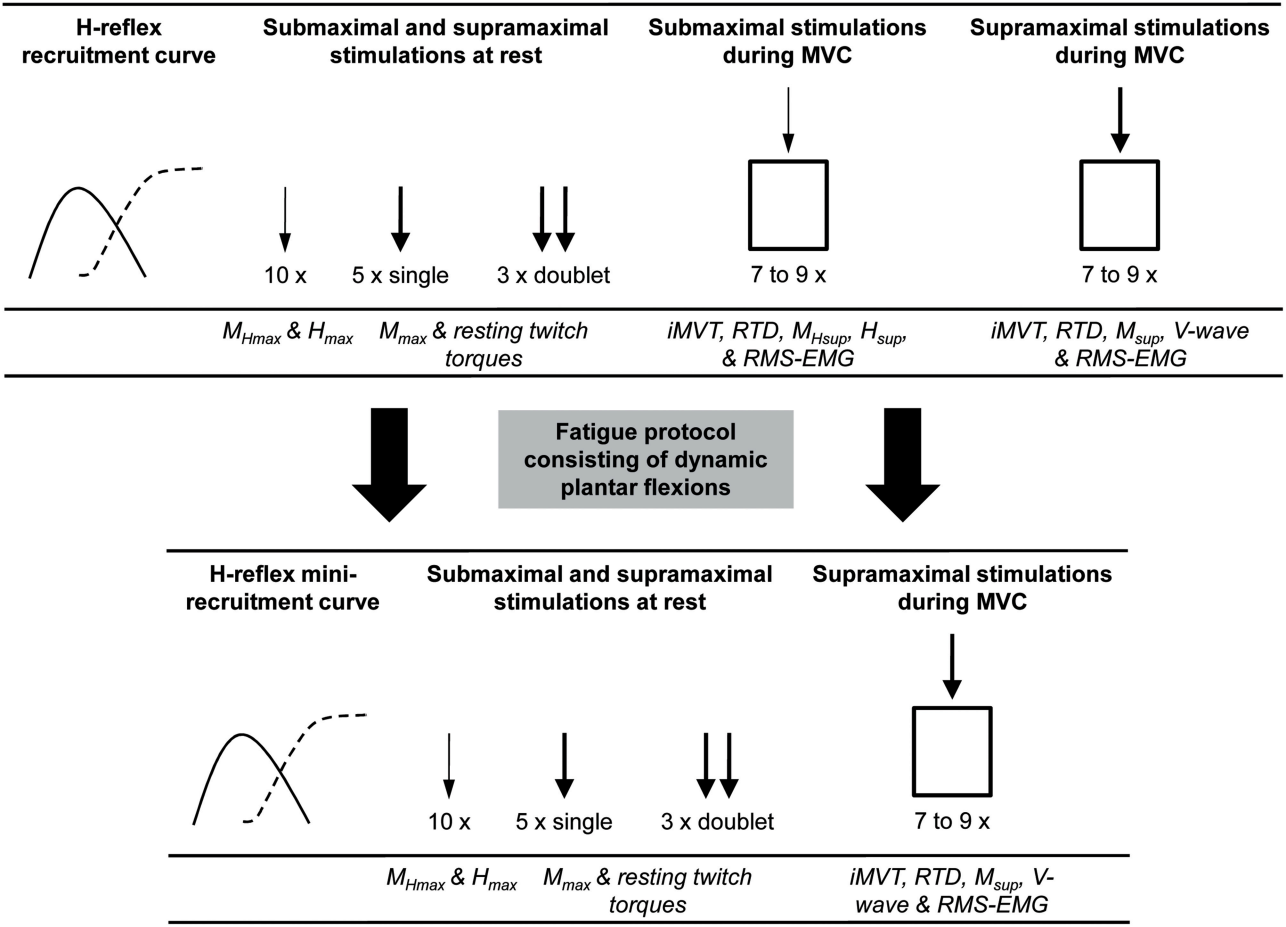
**An overview of the procedures carried out during neuromuscular testing and the extracted parameters**. The *thin arrow* indicates stimulation at H_max_ intensity and the *thick arrow* indicates stimulation at supramaximal intensity. M_Hmax_, submaximal M-wave evoked at H_max_ intensity; H_max_, maximal H-reflex; M_max_, maximal M-wave; M_Hsup_, submaximal M-wave evoked at H_sup_ intensity during iMVC; H_sup_, H-reflex during iMVC; M_sup_, maximal M-wave during iMVC; iMVT, isometric maximum voluntary torque; RTD, rate of torque development; RMS-EMG, root mean square of the EMG signal.

Neuromuscular function of the plantar flexors was measured prior to and after 8 weeks of either endurance training or a control period without the systematic training stimulus. The tests were performed under the same standardized conditions. To avoid H-reflex and M-wave potentiation no warm-up was utilized before neuromuscular testing (Folland et al., 2008). The measurements were performed on the triceps surae muscle and TA of the right leg. The participants were comfortably seated in a standardized position on a CYBEX NORM dynamometer during the testing sessions (Computer Sports Medicine®, Inc., Stoughton, MA, USA). Prior to neuromuscular testing, the subjects sat passively on the dynamometer for ~5 min in order to minimize potentiation effects from walking to the laboratory. Testing included different neuromuscular tests consisting of submaximal and supramaximal electrical stimulations of the posterior tibial nerve at rest and during iMVC (Figure 1).

Following this procedure subjects had to perform a standardized fatigue protocol consisting of dynamic plantar flexions in the isotonic mode of the CYBEX NORM dynamometer. Exercise intensity was 40% of iMVC strength and the subjects performed 35 contractions/min timed by a digital metronome. The fatigue protocol was stopped if the participants were not able to keep the metronome-guided contraction frequency or their individual range of motion for five consecutive contractions. After the fatigue protocol a further series of neuromuscular tests was performed (Figure 1). The same fatigue protocol, i.e., with the same load, repetitions and range of motion as before the training, was applied after the training intervention. With this approach we wanted to find out if endurance training is able to reduce the performance decrements associated with fatigue.

### Electrical stimulation

Electrical stimulation was performed as described previously (Behrens et al., 2015a,b). Briefly, the posterior tibial nerve was stimulated transcutaneously by two electrodes fixed in the popliteal fossa (cathode) and immediately distal to the patella on the anterior aspect of the knee (anode). A constant-current stimulator (Digitimer® DS7A, Hertfordshire, UK) in combination with a train/delay generator (Digitimer® DG2A, Hertfordshire, UK) allowed electrical stimulation (1 ms duration, 400 V maximal voltage). Identification of peak-to-peak maximal H-reflex (H_max_) and maximal M-wave (M_max_) of SOL was achieved by random stimulation (inter stimulus interval 7 s) with different current intensities. The H-reflex during iMVC (H_sup_) was elicited 2 s after torque onset during the plateau of the torque-time curve with H_max_ intensity to record the small M-wave preceding H_sup_ (M_Hsup_) and H_sup_. M_max_ responses at rest and during iMVC (M_sup_) as well as V-wave responses during iMVC were evoked with supramaximal stimulation intensity (140%).

Resting twitch torques were evoked using supramaximal single (1 ms duration, 400 V maximal voltage) and doublet stimuli (1 ms duration, 10 ms apart, 400 V maximal voltage). M_max_ amplitudes of TA were evoked by stimulating the peroneal nerve close to the fibular head with supramaximal stimuli. Following the fatigue protocol, a H-reflex mini-recruitment curve was established in order to adjust stimulation intensity for H-reflex, M-wave, V-wave and contractile properties testing as recommended (Rupp et al., 2010).

### EMG and torque recordings

Surface EMG was recorded using bipolar EMG Ambu® Blue Sensor N electrodes (2 cm diameter) firmly attached to the shaved, abraded and cleaned skin over the muscle bellies of SOL, medial gastrocnemius, lateral gastrocnemius and TA of the right leg and the tibia of the ipsilateral leg (reference electrode). A digital multimeter (MY-68, McVoice, Braunschweig, Germany) was used to measure the resistance between electrodes (<5 kΩ). Signals were amplified (2500×), band-pass filtered (10–450 Hz), and digitized with a sampling frequency of 5 kHz through an analog-to-digital converter (DAQ Card™-6024E, National Instruments, Austin, TX, USA). Both, the EMG and torque signals were sampled at 5 kHz and stored on a hard drive for later analysis with a custom built LABVIEW® based program (Imago, Pfitec, Endingen, Germany).

Torque signals were measured with a CYBEX NORM dynamometer (Computer Sports Medicine®, Inc., Stoughton, MA) equipped with a digital oscilloscope (HM1508, HAMEG Instruments, Germany) for instantaneous online visual feedback. Subjects were seated with their knees straight and their foot firmly attached to the adapter of the dynamometer using Velcro straps and a snowboard binding. Ankle and hip joint angles of 90 and 80° (0° = full extension) were maintained during the sessions and the ankle joint was aligned with the axis of the dynamometer. In order to prevent excessive movements and/or counter movements during recording of iMVC, straps across the thigh, waist and chest were used. For determination of isometric maximum voluntary torque (iMVT) subjects exerted maximal plantar flexions against the metal plate of the dynamometer for 3 s. A rest period of 1 min was allowed between the trials. The subjects performed three to five iMVC familiarization trials and testing was started once the coefficient of variance of three subsequent trials was below 5%. For H_sup_ and V-wave testing, seven to nine isometric maximal voluntary plantar flexions were performed, respectively (Behrens et al., 2015a,b).

### Data analysis

A gravity correction was applied to the torque signals and the mean of the signals recorded during the iMVC trials was used to calculate rate of torque development (RTD), iMVT and muscle activity. The average RTD over time intervals of 0–100 and 100–200 ms relative to the onset of contraction was calculated to give and index for explosive voluntary strength.

In addition, the root mean square of the EMG signal (RMS-EMG) in the same time intervals (0–100 and 0–200 ms relative to the onset of the EMG signals) was calculated to analyze muscle activation during the early phase of contraction. RMS-EMG during the iMVCs (RMS-EMG_iMVT_) was calculated over a 200 ms period at iMVT, i.e., 200 ms prior to the electrical stimulus. Muscle activities of SOL, medial gastrocnemius, lateral gastrocnemius and TA were normalized by dividing RMS-EMG by their respective M_max_ values (RMS-EMG/M_max_). RMS-EMG/M_max_ was averaged across SOL, medial gastrocnemius and lateral gastrocnemius to calculate triceps surae activation during the early phase of contraction and at iMVT (RMS-EMG_RTD_/M_max_ and RMS-EMG_iMVT_/M_max_, respectively). Torque and EMG onsets were identified manually according to the method of Tillin et al. (2010).

Peak-to-peak H_max_, M_Hmax_, M_max_, H_sup_, M_Hsup_, M_sup_, and V-wave amplitudes were averaged, respectively. The H_max_/M_max_-ratio, and H_sup_/M_sup_-ratio were calculated to detect modulations at the spinal level due to alterations in α-motoneuron excitability and/or presynaptic inhibition of primary muscle spindle afferents (Zehr, 2002). Furthermore, the M_Hmax_/M_max_-ratio and M_Hsup_/M_sup_-ratio were calculated to control for stimulation constancy, i.e., that the same proportion of α-motoneurons was activated by the electrical stimulation. The V/M_sup_-ratio was calculated to assess changes in the neural drive from spinal α-motoneurons to SOL (Aagaard et al., 2002; Duclay and Martin, 2005). Resting twitch torques were analyzed regarding the highest value of twitch torque signal (peak twitch torque) and were averaged afterwards.

### Statistical analysis

Twenty-two subjects completed the study. Data of these participants were collected successfully. Data were checked for normal distribution (Shapiro-Wilk test). In addition, the statistical analysis comprised an analysis of covariance (ANCOVA) with baseline measurement and gender entered as covariates (Vickers and Altman, 2001; Egbewale et al., 2014). This approach provides an estimate for the difference between groups which is the variable of interest in randomized controlled trials (Vickers, 2005a). The level of significance was established at *P* ≤ 0.05 and SPSS 20.0 (SPSS Inc., Chicago, IL, USA) was used for statistical analysis. Data obtained at baseline are presented as mean values ± standard deviations and those obtained after 8 weeks of training are given as adjusted means ± adjusted standard deviations. If appropriate, data are presented as difference between means (95% confidence interval).

## Results

Findings at baseline are given in Tables 2, 3. The participants of the intervention group performed the fatigue protocol with a load of 52.6 ± 7.3 N·m before and after the endurance training, while the control group executed the fatiguing task with a load of 56.9 ± 6.5 N·m (independent *t*-test: *P* = 0.181). The training group terminated exercise after 250.8 ± 124.3 repetitions before and after the training period and the control group performed 232.7 ± 106.1 repetitions before and after the same time without the training stimulus (independent *t*-test: *P* = 0.730).

**Table 2:** **Peak twitch torques, evoked potentials, maximum and explosive voluntary strength, normalized muscle activity (RMS-EMG/M_max_) during iMVC and the initial phase of contraction for the intervention (INT) and control group (CON) at baseline**.

**Parameter**	**Pre**		
	**INT**	**CON**	**Diff**.
**PEAK TWITCH TORQUE (N·m)**			
Supramaximal single	13.1 ± 2.5	14.9 ± 1.7	−1.8
Supramaximal doublet	25.4 ± 5.1	26.7 ± 2.5	−1.3
H-reflex intensity	10.1 ± 2.3	10.9 ± 1.9	−0.8
**EVOKED POTENTIALS**			
H_max_ SOL (mV)	3.68 ± 1.96	2.70 ± 0.92	0.98
M_max_ SOL (mV)	6.50 ± 2.23	5.00 ± 1.60	1.50
H_max_/M_max_ SOL	0.58 ± 0.19	0.57 ± 0.20	0.01
M_Hmax_/M_max_ SOL	0.16 ± 0.13	0.17 ± 0.14	−0.01
H_sup_ SOL (mV)	3.92 ± 1.83	3.24 ± 1.43	0.68
M_sup_ SOL (mV)	6.50 ± 1.94	5.98 ± 1.87	0.52
H_sup_/M_sup_ SOL	0.62 ± 0.12	0.52 ± 0.20	0.10
M_Hsup_/M_sup_ SOL	0.20 ± 0.10	0.17 ± 0.05	0.03
V-wave SOL (mV)	2.42 ± 1.07	2.15 ± 0.78	0.27
V/M_sup_ SOL	0.38 ± 0.13	0.38 ± 0.14	0.00
M_max_ MG (mV)	4.39 ± 1.97	5.01 ± 2.06	−0.62
M_max_ LG (mV)	5.83 ± 1.96	6.84 ± 2.34	−1.01
M_max_ TA (mV)	4.45 ± 1.54	4.28 ± 1.03	0.17
Isometric maximum voluntary torque (N·m)	93.2 ± 19.9	102.0 ± 16.2	−8.8
**RMS-EMG_iMVT_/M_max_**			
TS	0.038 ± 0.009	0.043 ± 0.007	−0.005
TA	0.022 ± 0.017	0.021 ± 0.009	0.001
**RATE OF TORQUE DEVELOPMENT (N·m·s^−1^)**			
0–100 ms	273.0 ± 71.6	293.9 ± 76.8	−20.9
100–200 ms	328.0 ± 64.9	355.8 ± 77.2	−27.8
**RMS-EMG_RTD_/M_max_**			
TS 0–100 ms	0.043 ± 0.015	0.044 ± 0.010	−0.001
TS 100–200 ms	0.047 ± 0.016	0.045 ± 0.008	0.002
TA 0–100 ms	0.021 ± 0.013	0.019 ± 0.007	0.002
TA 100–200 ms	0.026 ± 0.020	0.021 ± 0.010	0.005

*Diff., difference between means; H_max_, maximal H-reflex; M_max_, maximal M-wave; M_Hmax_, submaximal M-wave evoked at H_max_ intensity; H_sup_, H-reflex during iMVC; M_sup_, maximal M-wave during iMVC; M_Hsup_, submaximal M-wave evoked at H_sup_ intensity during iMVC; SOL, soleus; MG, medial gastrocnemius; LG, lateral gastrocnemius; TS, triceps surae; TA, tibialis anterior. Data are means ± standard deviations*.

**Table 3:** **Fatigue-induced percentage change in peak twitch torques, evoked potentials, maximum and explosive voluntary strength, normalized muscle activity (RMS-EMG/M_max_) during iMVC and the initial phase of contraction for the intervention (INT) and control group (CON) at baseline**.

**Parameter**	**Pre**		
	**INT**	**CON**	**Diff**.
**PEAK TWITCH TORQUE (%)**			
Supramaximal single	−5.2 ± 9.0	−9.4 ± 12.5	4.2
Supramaximal doublet	−7.0 ± 10.1	−8.3 ± 9.5	1.3
H-reflex intensity	3.2 ± 11.2	2.1 ± 15.7	1.1
**EVOKED POTENTIALS (%)**			
H_max_ SOL	13.5 ± 18.7	16.5 ± 21.6	−3.0
M_max_ SOL	1.4 ± 13.0	10.1 ± 19.4	−8.7
H_max_/M_max_ SOL	14.0 ± 20.1	12.0 ± 15.2	2.0
V-wave SOL	−37.0 ± 20.7	−27.1 ± 28.3	−9.9
M_sup_ SOL	−6.5 ± 15.0	−3.4 ± 10.5	−3.1
V/M_sup_ SOL	−32.6 ± 19.9	−25.1 ± 25.9	−7.5
Isometric maximum voluntary torque (%)	−13.6 ± 7.9	−8.6 ± 9.5	−5.0
**RMS-EMG_iMVT_/M_max_ (%)**			
TS	−26.5 ± 10.7	−27.8 ± 16.0	1.3
TA	−6.0 ± 6.2	−8.8 ± 7.5	2.8
**RATE OF TORQUE DEVELOPMENT (%)**			
0–100 ms	−23.7 ± 13.3	−24.6 ± 8.2	0.9
100–200 ms	−10.1 ± 11.2	−5.5 ± 10.3	−4.6
**RMS-EMG_RTD_/M_MAX_ (%)**			
TS 0–100 ms	−32.2 ± 14.3	−29.4 ± 13.5	−2.8
TS 100–200 ms	−27.0 ± 17.1	−22.2 ± 15.2	−4.8
TA 0–100 ms	−18.1 ± 9.6	−16.0 ± 7.2	−2.1
TA 100–200 ms	−13.8 ± 15.8	−12.7 ± 10.4	−1.1

*Diff., difference between means; H_max_, maximal H-reflex; M_max_, maximal M-wave; M_Hmax_, submaximal M-wave evoked at H_max_ intensity; H_sup_, H-reflex during iMVC; M_sup_, maximal M-wave during iMVC; M_Hsup_, submaximal M-wave evoked at H_sup_ intensity during iMVC; SOL, soleus; TS, triceps surae; TA, tibialis anterior. Data are means ± standard deviations*.

**Figure 2:**
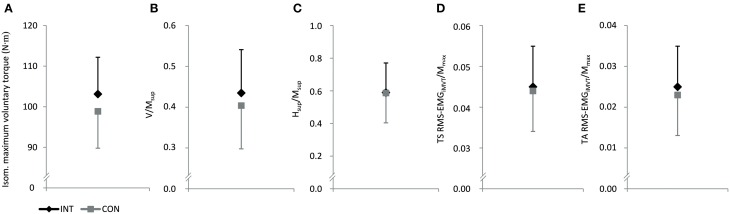
**Effect of endurance training (INT) on isometric maximum voluntary torque (A), normalized V-wave (V/M_sup_, B), normalized H-reflex during iMVC (H_sup_/M_sup_, C) and normalized muscle activity during iMVT (RMS-EMG_iMVT_/M_max_, D and E)**. CON, control group; TS, triceps surae; TA, tibialis anterior.

No significant differences between groups in iMVT [4.3 N·m (−4.0 to 12.5 N·m, *P* = 0.291, η^2^_*p*_ = 0.062)], V/M_sup_ [0.04 (−0.07 to 0.13, *P* = 0.508, η^2^_*p*_ = 0.025)], H_sup_/M_sup_ [0.00 (−0.21 to 0.22, *P* = 0.974, η^2^_*p*_ = 0.000)], triceps surae muscle activity [0.001 (−0.008 to 0.010, *P* = 0.791, η^2^_*p*_ = 0.004)], and TA muscle co-activity [0.002 (−0.008 to 0.012 N·m, *P* = 0.694, η^2^_*p*_ = 0.009)] were observed following the training (Figures 2A–E). Furthermore, the groups were not significantly different regarding the slope of the torque-time curve and the neural activation of muscles at the onset of contraction in the time intervals 0–100 and 100–200 ms (Table 4). The contractile performance as well as the remaining evoked potentials revealed no statistical difference between groups (Table 4).

**Table 4:** **Peak twitch torques and evoked potentials after training for the intervention (INT) and the control group (CON)**.

**Parameter**	**Post**			
	**INT**	**CON**	**Diff. (95% CI)**	***P***
**PEAK TWITCH TORQUE (N·m)**				
Supramaximal single	13.3 ± 1.8	12.9 ± 1.8	0.4 (−1.3 to 2.1)	0.626
Supramaximal doublet	24.6 ± 2.3	25.1 ± 2.3	−0.5 (−2.5 to 1.6)	0.629
H-reflex intensity	10.8 ± 1.9	10.0 ± 1.9	0.8 (−1.0 to 2.5)	0.363
**EVOKED POTENTIALS**				
H_max_ SOL (mV)	3.59 ± 1.12	3.49 ± 1.12	0.10 (−0.97 to 1.16)	0.853
M_max_ SOL (mV)	5.39 ± 1.60	6.35 ± 1.60	−0.96 (−2.54 to 0.63)	0.223
H_max_/M_max_ SOL	0.67 ± 0.13	0.61 ± 0.13	0.06 (−0.06 to 0.18)	0.285
M_Hmax_/M_max_ SOL	0.19 ± 0.09	0.18 ± 0.09	0.01 (−0.07 to 1.00)	0.696
H_sup_ SOL (mV)	3.80 ± 1.68	4.16 ± 1.68	−0.36 (−2.31 to 1.66)	0.723
M_sup_ SOL (mV)	6.56 ± 1.46	6.55 ± 1.46	0.01 (−1.31 to 1.34)	0.980
H_sup_/M_sup_ SOL	0.59 ± 0.21	0.59 ± 0.21	0.00 (−0.21 to 0.22)	0.974
M_Hsup_/M_sup_ SOL	0.14 ± 0.22	0.33 ± 0.22	−0.19 (−0.43 to 0.05)	0.114
V-wave SOL (mV)	2.69 ± 0.69	2.53 ± 0.69	0.16 (−0.47 to 0.78)	0.613
V/M_sup_ SOL	0.44 ± 0.11	0.40 ± 0.11	0.04 (−0.07 to 0.13)	0.508
M_max_ MG (mV)	4.73 ± 1.48	4.35 ± 1.48	0.38 (−0.95 to 1.71)	0.553
M_max_ LG (mV)	5.44 ± 1.40	6.37 ± 1.40	−0.93 (−2.20 to 0.34)	0.140
M_max_ TA (mV)	3.66 ± 1.42	4.01 ± 1.42	−0.35 (−1.63 to 0.92)	0.568
**RATE OF TORQUE DEVELOPMENT (N·m·s^−1^)**				
0–100 ms	299.3 ± 47.3	263.9 ± 47.3	35.4 (−7.1 to 78.0)	0.097
100–200 ms	380.5 ± 39.6	386.8 ± 39.6	−6.3 (−42.1 to 29.5)	0.716
**RMS-EMG_RTD_/M_max_**				
TS 0–100 ms	0.046 ± 0.010	0.044 ± 0.010	0.002 (−0.007 to 0.011)	0.650
TS 100–200 ms	0.049 ± 0.013	0.052 ± 0.013	−0.003 (−0.014 to 0.008)	0.582
TA 0–100 ms	0.025 ± 0.013	0.022 ± 0.013	0.003 (−0.008 to 0.013)	0.648
TA 100–200 ms	0.026 ± 0.013	0.025 ± 0.013	0.001 (−0.011 to 0.013)	0.892

*Diff. (95% CI), difference between means (95% confidence interval); H_max_, maximal H-reflex; M_max_, maximal M-wave; M_Hmax_, submaximal M-wave evoked at H_max_ intensity; H_sup_, H-reflex during iMVC; M_sup_, maximal M-wave during iMVC; M_Hsup_, submaximal M-wave evoked at H_sup_ intensity during iMVC; SOL, soleus; MG, medial gastrocnemius; LG, lateral gastrocnemius; TA, tibialis anterior. Data are adjusted means ± adjusted standard deviations*.

In addition, the change of crucial parameters following the same standardized fatigue protocol was analyzed before and after training to detect potential neural and/or muscular adaptations. These changes were not statistically different between groups with regard to iMVT, V/M_sup_ and contractile properties of the triceps surae assessed with doublet stimulation (Table 5). The same was true for the evoked potentials elicited at rest and during iMVC, the muscle activity during iMVC as well as for RTD and the neural activation of muscles at the onset of contraction (Table 5).

**Table 5:** **Fatigue-induced percentage change in peak twitch torques, evoked potentials, maximum and explosive voluntary strength, normalized muscle activity (RMS-EMG/M_max_) during iMVC and the initial phase of contraction after training for the intervention (INT) and control group (CON)**.

**Parameter**	**Post**			
	**INT**	**CON**	**Diff. (95% CI)**	***P***
**PEAK TWITCH TORQUE (%)**				
Supramaximal single	−0.3 ± 10.8	−8.3 ± 10.8	8.0 (−1.7 to 17.7)	0.101
Supramaximal doublet	−4.7 ± 12.7	−7.8 ± 12.7	3.1 (−8.4 to 14.5)	0.581
H-reflex intensity	−0.6 ± 15.1	−2.1 ± 15.1	1.5 (−12.4 to 15.5)	0.821
**EVOKED POTENTIALS (%)**				
H_max_ SOL	10.2 ± 22.8	26.2 ± 22.8	−16.0 (−36.1 to 4.0)	0.110
M_max_ SOL	10.2 ± 15.8	3.5 ± 15.8	6.7 (−7.9 to 21.3)	0.346
H_max_/M_max_ SOL	2.8 ± 23.9	24.0 ± 23.9	−21.2 (−43.3 to 0.9)	0.059
V-wave SOL	−23.4 ± 19.4	−34.7 ± 19.4	11.3 (−6.3 to 28.7)	0.195
M_sup_ SOL	−6.3 ± 11.7	−7.8 ± 11.7	1.5 (−8.9 to 12.0)	0.760
V/M_sup_ SOL	−18.8 ± 28.3	−26.6 ± 28.3	7.8 (−17.7 to 33.4)	0.527
Isometric maximum voluntary torque (%)	−4.4 ± 11.1	−10.4 ± 11.1	6.0 (−4.1 to 16.2)	0.229
**RMS-EMG_iMVT_/M_max_ (%)**				
TS	−23.8 ± 11.1	−24.1 ± 11.1	0.3 (−9.7 to 10.3)	0.953
TA	−18.4 ± 16.3	−21.4 ± 16.3	3.0 (−11.9 to 17.9)	0.675
**RATE OF TORQUE DEVELOPMENT (%)**				
0–100 ms	−17.7 ± 16.2	−17.7 ± 16.2	0.0 (−14.7 to 14.4)	0.983
100–200 ms	−3.7 ± 12.6	−12.7 ± 12.6	9.0 (−2.4 to 20.5)	0.114
**RMS-EMG_RTD_/M_max_ (%)**				
TS 0–100 ms	−28.8 ± 17.2	−24.4 ± 17.2	−4.4 (−19.9 to 11.1)	0.560
TS 100–200 ms	−23.4 ± 17.4	−22.9 ± 17.4	−0.5 (−16.2 to 15.2)	0.950
TA 0–100 ms	−21.6 ± 16.1	−23.4 ± 16.1	1.8 (−13.0 to 16.6)	0.800
TA 100–200 ms	−18.5 ± 17.0	−16.6 ± 17.0	−1.9 (−17.4 to 13.5)	0.794

*Diff. (95% CI), difference between means (95% confidence interval); H_max_, maximal H-reflex; M_max_, maximal M-wave; M_sup_, maximal M-wave during iMVC; SOL, soleus; TA, tibialis anterior. Data are adjusted means ± adjusted standard deviations*.

## Discussion

The purpose of this randomized controlled study was (i) to analyze neuromuscular adaptations of the plantar flexors after short-term (8 weeks) cycling endurance training and (ii) to assess selected variables, representing neural and muscular function of the plantar flexors, after a defined fatigue protocol with the same load, repetitions and range of motion before and after the training period. The results indicate that cycling endurance training did not lead to a significant change in any variable of interest. Neither normalized evoked potentials elicited at rest and associated peak twitch torques, isometric explosive and maximum voluntary strength nor muscle activation at the onset of contraction and during iMVC were significantly different between the training and control group after a period of either cycling endurance training or no systematic cycling endurance training, respectively. Furthermore, no differences between groups were observed regarding the normalized evoked potentials elicited during iMVC and the changes of selected variables, representing neural and muscular function of the plantar flexors, after defined fatiguing exercise.

Previously published studies on the effect of endurance training on neuromuscular function of the plantar flexors have shown that stretch reflexes and H-reflexes of SOL were significantly increased after training (Perot et al., 1991; Vila-Cha et al., 2012b). These results conflict with the outcome of this study that could not confirm an increased recruitment of α-motoneurons via the Ia afferent pathway after a period of endurance training. Reasons for the discrepancy between the outcomes of the cited studies and our results could be that the investigated subjects responded differently to the training stimulus and/or the difference in the kind of endurance training. However, our subjects were recreationally active just like the subjects in the cited studies and it has been previously shown that an endurance training program performed on a cycle ergometer is effective in modulating spinal reflex responses (Vila-Cha et al., 2012b). Thus, another more probable explanation could be that both Perot et al. (1991) and Vila-Cha et al. (2012b) had not included a control group in their study design in order to compare the results of the endurance training group to individuals without the endurance training stimulus. In order to analyze our data as done in the studies mentioned, we have applied a paired statistical test (dependent *t*-test) for comparison of the normalized H-reflex at rest (H_max_/M_max_) before and after the training. The analysis yielded a significant increase in H_max_/M_max_-ratio (*P* = 0.047). Based on this result, we could also state that endurance training increases the normalized H-reflex. However, if we use an analysis of covariance (ANCOVA) with baseline measurement and gender entered as covariates, which is supposed to be the appropriate statistical test for analyzing the effect of an intervention compared to a control condition in randomized controlled trials (Vickers and Altman, 2001; Vickers, 2005a,b; Egbewale et al., 2014), no significant group difference can be revealed. Even Perot et al. (1991) observed that 75% of their investigated subjects showed an increased stretch reflex and H-reflex excitability after the endurance training whereas the remaining 25% of the subjects revealed no change or even a decrease in these parameters. Thus, it is rather likely that short-term endurance training does not necessarily enhance stretch and H-reflex responses. Based on our results and statistical analysis, an increased H-reflex excitability and/or changed presynaptic inhibition of Ia afferents after short-term cycling endurance training cannot be confirmed. Although it has been shown that endurance trained athletes (10–14 h endurance training per week for several years) have higher normalized H-reflexes than non-trained individuals (Maffiuletti et al., 2001), our data indicate that short-term endurance training performed on a cycle ergometer seems not to alter the recruitment threshold of α-motoneurons to Ia afferent input at rest. It can be assumed that a longer time of regular aerobic exercise is necessary to induce alterations in reflex responses. Moreover, as the contribution of stretch reflexes to overall muscle activity is greater during running, it is not unlikely that running training has stronger effects on spinal reflex responses than cycling training.

To the best of our knowledge H-reflex modulation during iMVC after a period of cycling endurance training was not tested before. In the present study we have found that the H-reflex evoked during iMVC (H_sup_/M_sup_) was unchanged after training. Therefore, short-term endurance training on a cycle ergometer is probably not able to alter the responsiveness of spinal a-motoneurons to Ia afferent input during isometric maximum voluntary strength tasks of the plantar flexors.

The training regimen had no effect on isometric explosive and maximum voluntary strength as well as muscle activation at the onset of contraction (RMS-EMG_RTD_/M_max_) and during iMVC (V/M_sup_, RMS-EMG_iMVT_/M_max_). These results are in accordance with the outcome of a study by Vila-Cha et al. (2012b). The authors have shown that short-term endurance training on a cycle ergometer did not alter iMVC strength and neural activation of the plantar flexors assessed with the V-wave. Data of a recently published study on the effect of endurance training (running) on voluntary activation of the knee extensors support this view (Zghal et al., 2014).

The change in the resistance to fatigue following a period of endurance training was previously tested by sustained isometric contractions with a defined percentage of iMVC strength until task failure. With this approach, it has been shown that time to task failure increases following endurance training (Vila-Cha et al., 2012a,b). However, the time to task failure does not provide information regarding neural and muscular contributions to the improved resistance to fatigue. Therefore, we applied the same dynamic fatigue protocol, i.e., with the same load, repetitions and range of motion, before and after the cycling endurance training period and assessed the changes in neuromuscular function of the plantar flexors. With this approach, we wanted to find out if cycling endurance training is able to reduce the performance decrements associated with fatigue. Our data show that the percentage changes of these parameters following fatiguing exercise were not significantly different between groups after the training. However, the results indicate that the fatigue-induced reduction in iMVC strength, normalized V-wave and peak twitch torque in response to the same standardized fatigue protocol tended to be lower in the endurance-trained group compared to controls, but this change did not reach statistical significance (Table 5). Unfortunately, we have not measured cycling performance before and after the training. However, numerous studies have shown that cycling endurance training with a similar intensity, as used in the present study, leads to an increase in endurance performance and corresponding physiological adaptations even in recreational active subjects (Ready and Quinney, 1982; Denis et al., 1984; Hickson et al., 1985; Coggan et al., 1990; Levine et al., 1992). We have compared the changes in iMVC strength due to the fatigue protocol before and after the training with a paired *t*-test (*P* = 0.033) indicating a significant reduction in the performance decrements. Based on this result, we could state that the performed cycling endurance training increased the fatigue resistance with regard to our specific fatigue protocol. However, if we use an analysis of covariance (ANCOVA) with baseline measurement and gender entered as covariates, which is supposed to be the appropriate statistical test for analyzing the effect of an intervention compared to a control condition in randomized controlled trials (Vickers and Altman, 2001; Vickers, 2005a,b; Egbewale et al., 2014), no significant group difference can be revealed. However, in this context it is noteworthy to illustrate the limitations of the present study regarding this issue: (i) we have not measured the effect of our training regimen on maximal oxygen uptake and cycling endurance performance, (ii) we have used a relative low training frequency of two times a week and (iii) SOL muscle activation during cycling is relative low (Rouffet and Hautier, 2008) and it might be that the training stimulus is not sufficient to induce neuromuscular adaptations in the SOL muscle of recreational active subjects.

In contrast to the outcome of previously published studies, the results of our randomized controlled trial indicate that short-term endurance training on a cycle ergometer seems not to alter the recruitment threshold of α-motoneurons to Ia afferent input and/or the extent of presynaptic inhibition of Ia afferents at rest. Furthermore, we have tested the responsiveness of spinal α-motoneurons to Ia afferent input during iMVC for the first time and have not found a modulation in α-motoneuron excitability and/or presynaptic inhibition of Ia afferents. The training regimen had no effect on isometric explosive and maximum voluntary strength as well as muscle activation at the onset of contraction (RMS-EMG_RTD_/M_max_) and during iMVC (V/M_sup_, RMS-EMG_iMVT_/M_max_). Changes in iMVC strength, neural activation (V/M_sup_) and muscles' contractile properties after the same standardized fatigue protocol applied before and after the training were not significantly different between groups.

Future research on the effect of short-term endurance training should analyze the impact of different training frequencies and types of activity, e.g., running, swimming, cycling, on evoked reflex responses and neuromuscular function. It is likely that changes in evoked spinal reflex responses depend on the kind of activity and its reliance on the stretch-shortening cycle. From this functional point of view, activities like running are more predestined to induce adaptations at the spinal level.

### Conflict of interest statement

The authors declare that the research was conducted in the absence of any commercial or financial relationships that could be construed as a potential conflict of interest.
